# Long-term protection of hepatitis B vaccine in HIV-infected patients

**DOI:** 10.1186/1471-2334-14-S2-O7

**Published:** 2014-05-23

**Authors:** R Biekre, N Ben Lasfar, N Viget, M Valette, V Baclet, T Huleux, E Senneville, F Ajana

**Affiliations:** 1 Gustave Dron Hospital, Infectious Diseases Department, Tourcoing, France

## Aim

Many studies reported in HIV-positive patients 50% of hepatitis B vaccine response with a duration of protection between 24 and 36 months. The aim of our study is to determine the long term protection in our HIV patients who responded to a standard immunization schedule.

## Materials and methods

Our database NADIS allows us to collect information from March 1986 to October 2013 (gender, age, CD4 count, HIVRNA and Hepatitis B surface antibody titers = HBs Ab > 10 IU / L.)

Patients were included gradually after 3 Engerix B 20 hepatitis B vaccines at day 1, one month and 6 months later, conferring a rate of HBs ab > 10 IU /L. Then we observed the duration of protection from the initial response.

## Results

Among 403 HIV patients who responded to HBV, 298 were men (74%) vs 105 women (26%). The median age was 36 years. We observed a progressive decline in HBs ab titer until <10 IU/ L. At 24 months 74.7% (n=223) men remained protected vs 79.2% (n=83) women. At 60 months. 33.1% (n=99) men lost their HB protection vs 27.7% (n=29) women (LR = 0,11 NS).

At 84 months and over 120 months, 34.7% (n=103) and 39.1% (n=117) men lost their HB protection vs 35.8% (n=38) and 38.2% women (n=40).

## Conclusions

Long term HBV protection was observed in the majority of our HIV patients. One third had lost their HBs ab after 2 years, and require HB boost injections. We are thinking of using an adjuvanted HB vaccine as a booster to provide a sustained protection. We will also study factors associated with the loss of HBV protection.

**Figure 1:**
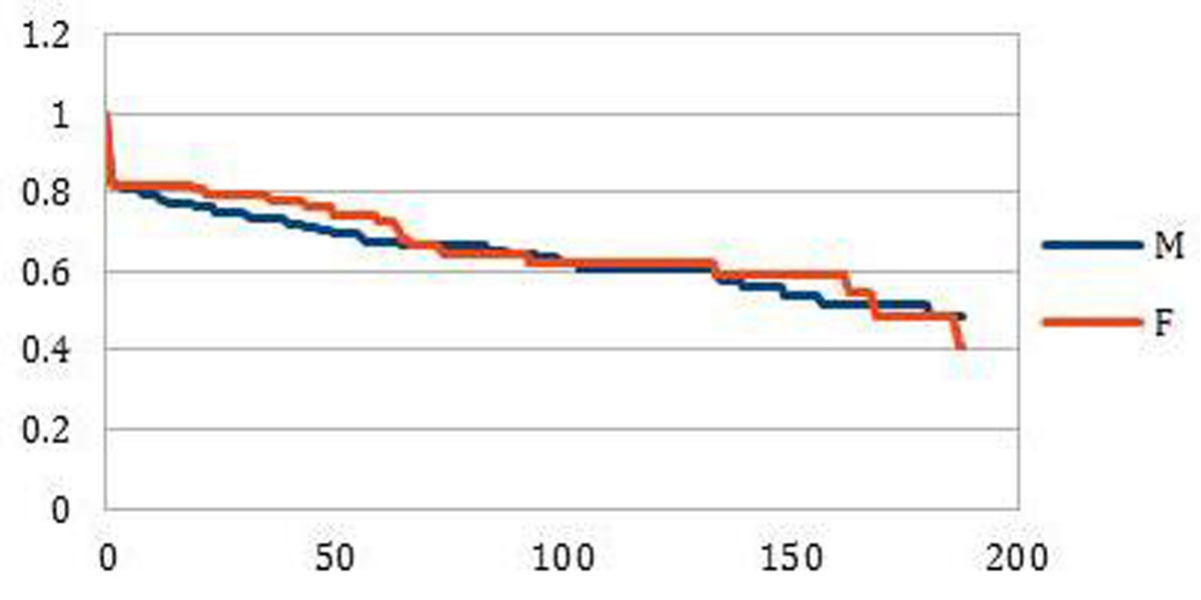
Duration of HBV protection (months)

